# Involvement of RpoN in Regulating Motility, Biofilm, Resistance, and Spoilage Potential of *Pseudomonas fluorescens*

**DOI:** 10.3389/fmicb.2021.641844

**Published:** 2021-05-31

**Authors:** Xiaoxiang Liu, Yifan Ye, Yin Zhu, Lifang Wang, Leyang Yuan, Junli Zhu, Aihua Sun

**Affiliations:** ^1^School of Basic Medical Sciences and Forensic Medicine, Hangzhou Medical College, Hangzhou, China; ^2^Zhejiang Museum of Natural History, Hangzhou, China; ^3^College of Food Science and Biotechnology, Zhejiang Gongshang University, Hangzhou, China

**Keywords:** *Pseudomonas fluorescens*, motility, biofilm, resistance, spoilage potential, RNA-Seq

## Abstract

*Pseudomonas fluorescens* is a typical spoiler of proteinaceous foods, and it is characterized by high spoilage activity. The sigma factor RpoN is a well-known regulator controlling nitrogen assimilation and virulence in many pathogens. However, its exact role in regulating the spoilage caused by *P*. *fluorescens* is unknown. Here, an in-frame deletion mutation of *rpoN* was constructed to investigate its global regulatory function through phenotypic and RNA-seq analysis. The results of phenotypic assays showed that the *rpoN* mutant was deficient in swimming motility, biofilm formation, and resistance to heat and nine antibiotics, while the mutant increased the resistance to H_2_O_2_. Moreover, the *rpoN* mutant markedly reduced extracellular protease and total volatile basic nitrogen (TVB-N) production in sterilized fish juice at 4°C; meanwhile, the juice with the *rpoN* mutant showed significantly higher sensory scores than that with the wild-type strain. To identify RpoN-controlled genes, RNA-seq-dependent transcriptomics analysis of the wild-type strain and the *rpoN* mutant was performed. A total of 1224 genes were significantly downregulated, and 474 genes were significantly upregulated by at least two folds at the RNA level in the *rpoN* mutant compared with the wild-type strain, revealing the involvement of RpoN in several cellular processes, mainly flagellar mobility, adhesion, polysaccharide metabolism, resistance, and amino acid transport and metabolism; this may contribute to the swimming motility, biofilm formation, stress and antibiotic resistance, and spoilage activities of *P*. *fluorescens*. Our results provide insights into the regulatory role of RpoN of *P*. *fluorescens* in food spoilage, which can be valuable to ensure food quality and safety.

## Introduction

*Pseudomonas* spp. are Gram-negative, rod-shaped, ubiquitous microorganisms with simple nutritional needs (Remenant et al., 2015). They largely contribute to the food spoilage process, and among the genus, *Pseudomonas fluorescens* is a typical spoiler of proteinaceous raw foods stored under aerobic refrigerated conditions, especially aerobically chill-stored seafood (Xie et al., 2018), meat (Doulgeraki and Nychas, 2013), and milk (Polêto et al., 2019). The major cause of food spoilage is microbial growth and metabolism leading to degradation of polymers or off-odors and off-flavors that alter the sensory quality of food (Gram and Dalgaard, 2002). Several factors can contribute to the spoilage potential of bacteria. Motility is required for colonization and competition in food systems (Wang et al., 2018); biofilms can form on surfaces of various foods or food industry equipment and are often difficult to be removed by normal sanitation procedures (Galié et al., 2018); and the bacterial resistance to stress conditions during food preparation and processing and to antibiotics during agricultural production leads to persistent contamination in food systems (Verraes et al., 2013; Liu X. et al., 2018). Understanding how the bacterium regulates its spoilage ability is vital; however, the knowledge related to the regulatory mechanisms of spoilage is still limited.

Bacteria can control their colonization and growth in specific environments through gene transcriptional regulation. The initiation of transcription is catalyzed by core RNA polymerase associated with a sigma factor that recognizes the specific promoter elements and decides the transcription of specific genes (Feklístov et al., 2014). The alternative sigma factor RpoN recognizes a characteristic −24/−12 promoter and requires an associated activator to initiate the transcription of specific genes (Zhang et al., 2016). RpoN is commonly found in Gram-negative and Gram-positive species. It plays a role in nitrogen metabolism, such as the secretion of extracellular protease (Hao et al., 2013; Lloyd et al., 2017), amino acid catabolism, and ammonia assimilation (Riordan and Mitra, 2017). Additionally, RpoN is implicated in the control of genes essential to virulence, including those involved in the adherence (Riordan and Mitra, 2017), secretion (Shao et al., 2018), biofilm formation (Liu Y. et al., 2018), and resistance to both of antimicrobials (Hall et al., 2019) and biological stressors (Xu et al., 2019) in many pathogens.

RpoN regulons have been characterized by microarrays or RNA-seq in many bacteria. The deletion of *rpoN* has been shown to alter the RNA transcript levels of 103 genes in *Escherichia coli* O157, including the genes required for glutamate-dependent acid resistance and type III secretion system (Riordan et al., 2010). Transcriptomic profiling revealed that 562 genes in *Pseudomonas protegens* H78 were significantly upregulated, and 502 genes were downregulated in the *rpoN* deletion mutant compared with the wild-type strain (Liu Y. et al., 2018), and these genes were mainly involved in flagellar biogenesis and assembly, bacterial mobility, biofilm formation, antibiotic biosynthesis, secretion systems, and carbon utilization. In *Pseudomonas aeruginosa*, blocking RpoN by a *cis*-acting peptide reduced transcription of about 700 genes, including genes related to motility, protease secretion, pyocyanin and pyoverdine production, rhamnolipid production, and biofilm formation. The genes and phenotypes controlled by RpoN depend on the genetic background and growing conditions of bacteria (Lloyd et al., 2017).

The involvement of RpoN in nitrogen metabolism and virulence implies that RpoN is likely to control the spoilage ability of *P*. *fluorescens* in proteinaceous food systems. Our previous work showed that the sigma factor RpoS mainly regulated the resistance and quorum sensing of *P*. *fluorescens* UK4. The production of extracellular proteases and TVB-N by the *rpoS* mutant in sterilized fish juice was lower than the production by the wild-type strain (Liu X. et al., 2018). Recently, Tang et al. (2019) indicated that the deletion of the quorum-sensing system LuxI/LuxR of *P*. *fluorescens* PF07 mainly inhibited the biofilm and resistance formation but did not affect the TVB-N production in fish juice. Therefore, RpoN may be another important regulator controlling the spoilage activity of *P*. *fluorescens*.

In this study, we explored the role of RpoN in the spoilage process of *P*. *fluorescens* UK4. An in-frame deletion mutant of *rpoN* was constructed to determine the involvement of RpoN in regulating motility, biofilm, resistance, and spoilage activity. Moreover, RNA-seq-dependent transcriptomics analysis was performed to elucidate the spoilage regulatory mechanism of RpoN in *P*. *fluorescens*.

## Materials and Methods

### Strains and Growth Conditions

The bacterial strains and the plasmids used in this study are presented in Supplementary Table 1. Unless otherwise stated, *P*. *fluorescens* and *E*. *coli* strains were grown in Luria-Bertani (LB) medium at 28 and 37°C, respectively. *E*. *coli* β2163, a diaminopimelic acid (DAP) auxotroph, was grown on LB plates with 0.3 mM DAP.

### Construction of *rpoN* In-Frame Deletion Mutant

The genome annotation of *P*. *fluorescens* UK4 showed that one open reading frame (ORF) (HZ99_RS12105) was *rpoN*, and the *rpoN* in-frame deletion mutant was constructed by double-crossover allelic exchange according to the method described previously (Liu X. et al., 2018). All primers used in the mutant construction were listed in Supplementary Table 1. Briefly, a 424-bp DNA fragment containing the upstream region of *rpoN* was amplified from chromosomal DNA via polymerase chain reaction (PCR) using primers *rpoN*-MF and *rpoN*-MR1. A 648-bp DNA fragment containing the downstream region of *rpoN* was amplified using primers *rpoN*-MF2 and *rpoN*-MR2. There was a 42-bp overlap in the two fragments, and both were purified and fused in a subsequent PCR using primers *rpoN*-MF1 and *rpoN*-MR2. The fused segment, representing a deletion of positions 4–1473 bp of the *rpoN* ORF (1494 bp), was ligated into the suicide vector pLP12Tc using ClonExpress II One Step Cloning Kit (Vazyme, China). The resulting plasmid pLP12Tc-*rpoN* was transferred into competent *E. coli* DH5α, and then was extracted and transformed into *E*. *coli* β2163 via electroporation. The plasmid was transferred into *P*. *fluorescens* UK4 through conjugation. The transconjugants with the plasmid integrated into the chromosome were selected on an LB agar medium containing 24 μg/mL tetracycline and 0.3% D-glucose at 28°C. The second crossover mutants were screened on LB agar supplemented with 0.4% L-arabinose. The UK4 Δ*rpoN* mutant was confirmed via PCR using two external primers, *rpoN*-TF and *rpoN*-TR, anchored upstream and downstream of the *rpoN* gene. The deletion mutant was subsequently confirmed by DNA sequencing.

### Swimming Motility

Swimming motility assays were performed following a previously reported procedure (Tang et al., 2019). Briefly, the overnight cultures of UK4 and the *rpoN* mutant (5 μL each) were spotted on swimming agar plates (1% tryptone, 0.5% NaCl, and 0.3% agar) and cultured at 28°C for 1, 2, 3, and 4 days. Bacteria migrated through the agar from the center of the plate toward the periphery. The diameters of the migrating zones were measured, and images of the plates were daily captured.

### Crystal Violet Assay for Biofilm in Microplates

Biofilm formation in microplates was quantified via crystal violet staining according to the method previously described (Dueholm et al., 2013), with a little modification. Overnight cultures of UK4 and the *rpoN* mutant were diluted at a 1:1000 ratio in fresh sterile LB or tryptone broth (1% tryptone). The dilutions were transferred into 96-well microplates at a volume of 200 μL per well. The plate was then incubated for 6, 12, 24, and 48 h at 28°C with shaking. The wells were aspirated and washed with running distilled water, and were then dried in air. The biofilms were stained for 15 min with 250 μL of 1% crystal violet solution. The wells were washed thoroughly with running water and allowed to dry in air. Crystal violet quantification was performed by solubilizing the crystal violet with ethanol for 30 min, and measuring the absorbance at 620 nm. Eight individual samples were assessed for each strain at each time point, and the experiment was repeated at least twice.

### Congo-Red Assay and Transmission Electron Microscopy for Macrocolony Biofilm Analysis

Macrocolony biofilms were observed using the previously reported method (Liu et al., 2019). The overnight cultures of UK4 and the *rpoN* mutant (5 μL each) were spotted on Congo-red plates (1% tryptone, 1% agar, 20 μg/mL Congo red, and 10 μg/mL Coomassie brilliant blue G250). The plates were incubated at 28°C for up to 7 days to observe the macrocolony morphology. For transmission electron microscopy (TEM) analysis, the 7-day-old bacterial colonies were gently scraped from a tryptone plate (1% tryptone, 1% agar) and fixed in a solution of 2.5% glutaraldehyde for 2 h at room temperature. After the macrocolony biofilms were postfixed, dehydrated, embedded in TAAB resin, ultrathin-sectioned, and stained, they were observed under a transmission electron microscope (Hitachi H-600, Japan).

### Stress Resistance

The stress resistances of UK4 and the *rpoN* mutant were assayed as described in a previous study (Liu X. et al., 2018). Briefly, bacterial cells at the stationary phase (OD_600_ ≈ 1.5) were collected and diluted with 0.1 M phosphate buffer (pH 7.0) to an initial population of 10^6^–10^7^ cfu (colony forming units)/mL. The dilutions were exposed to several stress conditions, including 10 mM H_2_O_2_, 47°C, 12% (v/v) ethanol, and 20% (m/v) NaCl. After exposure for 15, 30, and 45 min, viable counts were carried out to monitor viability.

### Antibiotic Resistance

Disk diffusion testing was used to determine the antibiotic susceptibility of UK4 and the *rpoN* mutant according to the guidelines of National Committee for Clinical Laboratory Standards [NCCLS] (2003). The following 19 antimicrobial disks were used: streptomycin (10 U), cefepime (30 μg), rifampicin (10 μg), nalidixic acid (30 μg), ciprofloxacin (30 μg), cefotaxime (30 μg), sultamicillin (15 μg), fosfomycin (15 μg), chloramphenicol (10 μg), vancomycin (30 μg), norfloxacin (30 μg), azithromycin (200 μg), erythromycin (10 μg), tetracycline (30 μg), neomycin (5 μg), kanamycin (30 μg), gentamicin (5 μg), cephalexin (10 μg), and penicillin (30 μg). Bacterial suspensions at a density adjusted to a 0.5 McFarland turbidity standard were spread onto Mueller-Hinton agar plates, and the antimicrobial disks were placed onto these plates, which were then incubated at 28°C for 24 h. The diameters of inhibitory zones were measured and recorded.

### Spoilage Potential in Sterile Fish Muscle Juice

The spoilage potential of UK4 and the *rpoN* mutant were assayed in sterile fish juice (*Pseudosciaena crocea*) at 4°C, which was prepared according to the method by Dalgaard (1995). Overnight cultures of the strains were diluted in 0.9% NaCl and were inoculated into fish juice to achieve an inoculation mixture containing 10^5^–10^5^.^5^ cfu/mL. All batches of inoculated fish juice were stored at 4°C for 7 days. The juice samples were analyzed to daily determine the sensory score, total viable count (TVC), extracellular protease activity, and total volatile basic nitrogen (TVB-N). Regarding sensory assessment, the juice samples were evaluated by seven trained panelists. The appearance, odor, and general acceptability of the juice samples were scored using a nine-point hedonic scale. A sensory score of five was considered the borderline of acceptability (Zhu et al., 2016). For the TVC assay, tenfold dilutions of juice samples were poured in plate count agar, and viable counts were carried out after 48 h of incubation at 28°C. Extracellular protease activity was assayed using previously reported method (Liu X. et al., 2018). One unit of protease activity was defined as the amount of enzyme causing the generation of 1.0 μg tyrosine per minute under the specified test condition. The TVB-N (mg N/mL) was detected via steam distillation with a FOSS Kjeltec 8400 automatic nitrogen-determination apparatus (Foss, Denmark) (Liu X. et al., 2018).

### RNA Extraction

Overnight cultures of UK4 and the *rpoN* mutant were inoculated into fish juice in triplicates and were cultured at 4°C for 6 days as mentioned above. Bacterial cells were harvested by centrifugation at 4°C and were then immediately frozen in liquid nitrogen for RNA isolation. The total RNA was isolated with an RNAprep Pure Cell/Bacteria Kit (Tiangen, China). The RNA concentration and purity were checked using a NanoDrop spectrophotometer (Thermo, United States), and the RNA integrity was assessed using agarose gel electrophoresis and Agilent 2100 bioanalyzer (Agilent, United States).

### RNA-seq and Data Analysis

The RNA-seq and the data analysis were performed according to a previously reported procedure (Liu et al., 2019). Briefly, the rRNA from the total RNAs was removed using a Ribo-Zero Magnetic Kit (Epicentre, United States). Strand-specific RNA sequencing libraries were prepared with NEBNext Ultra Directional RNA Library Prep Kit for Illumina (NEB, United States) following the manufacturer’s recommendations. Three independent libraries were constructed for each of the RNA-seq samples. Following validation with the Agilent Bioanalyzer 2100 system (Agilent, United States), the cDNA library was sequenced on an Illumina platform using 150-bp pair-end mode (Illumina, United States). After the raw reads were filtered, the clean reads were aligned to the reference *P*. *fluorescens* UK4 genome (Dueholm et al., 2014). The thresholds for significantly different expression levels were | log_2_ fold change| ≥ 1 and adjusted *p*-value (padj) ≤ 0.05. Putative operons were identified based on the criteria that every ORF was in the same orientation, every ORF had the same trend in differential expression, and the intergenic region between two adjacent ORFs was <250 bp (Schuster et al., 2004; Liu et al., 2019). The KEGG pathway annotations for differentially expressed genes (DEGs) were performed using the KEGG database^1^. A two-tailed Fisher’s exact test was employed to test the enrichment of the DEGs against all identified genes. KEGG pathways with *p*-values < 0.05 were considered significant.

### Quantitative Realtime PCR (qRT-PCR)

The RNA-seq results were further confirmed by qRT-PCR according to the method described by Liu et al. (2019). The RNA samples for RNA-seq were used in the qRT-PCR assays, and were reversely transcribed using a hexamer primer and SuperScript III First-Strand Synthesis SuperMix (Invitrogen, United States). The primers for 20 genes downregulated in the *rpoN* mutant were designed and are listed in Supplementary Table 2. The 16S rRNA gene was used as an internal control for sample normalization. The qRT-PCR was conducted on a CFX384 Touch real-time PCR detection system (Bio-Rad, United States) using Power SYBR1 Green PCR Master Mix (Applied Biosystems, United States). The specificity of the amplification was evaluated through the melting curve analysis of amplification products. The relative expression was calculated using the 2^−ΔΔCt^ method (Livak and Schmittgen, 2001). Two biological replicates were performed, and samples were run in triplicate for qRT-PCR.

## Results

### Construction of *rpoN* Mutant by In-Frame Deletion

The *rpoN* in-frame deletion mutant was constructed by double-crossover allelic exchange to investigate the role of RpoN in *P. fluorescens* UK4. The ORF of the *rpoN* gene was 1494 bp. The core portion from bp 4 to 1473 of the ORF was deleted, and the *rpoN* mutant was verified via PCR (Supplementary Figure 1) and sequencing. We tried to construct the complemented strain of this mutant using different methods with broad-host range plasmids pVLT33 (de Lorenzo et al., 1993) or pBAD33 (Guzman et al., 1995), but failed to obtain the right complemented strain of *P*. *fluorescens* UK4. Probably, the two plasmids we used are not suitable expression vectors for UK4.

### RpoN Is Require for Swimming Motility

**FIGURE 1:**
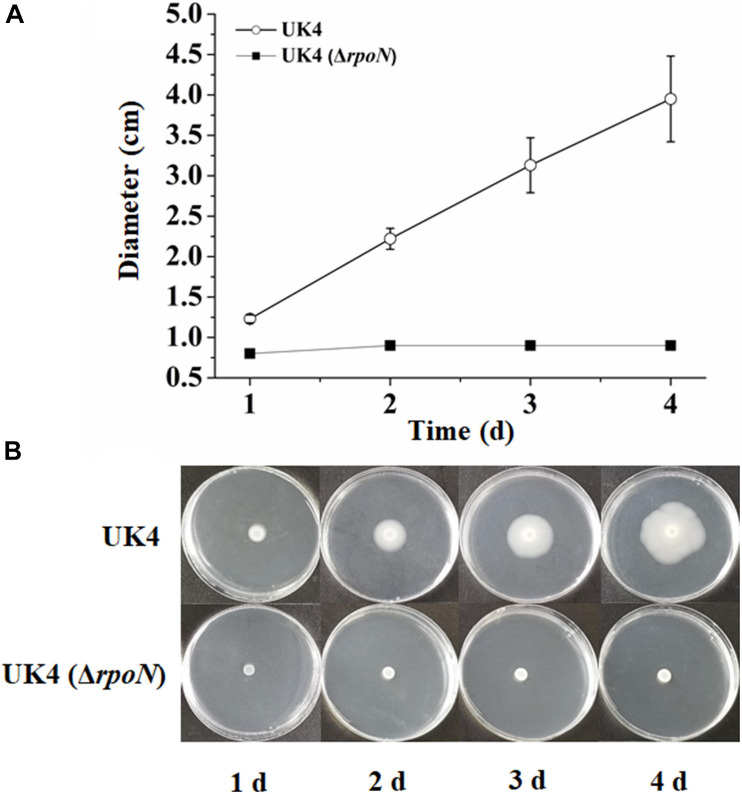
Swimming motility of *P*. *fluorescens* wild-type strain UK4 and the mutant strain Δ*rpoN*. **(A)** Swimming diameter of UK4 and Δ*rpoN*. Data are expressed as mean ± SD of six independent experiments. **(B)** Swimming of UK4 and Δ*rpoN* on swimming-agar plates for 4 days.

Swimming motility is a mode of bacterial movement powered by rotating flagella (Berg, 2003). The swimming motilities of UK4 and the *rpoN* mutant were assayed by measuring the diameter of the migrating zone on the swimming agar plates for 4 days. The agar concentration of the swimming agar plates is far below that of the normal agar plates, and the strain with active flagella can swim around and form a migrating zone in the swimming agar plates. As shown in Figure 1, the migrating zones of the wild-type strain were remarkably larger than those of the mutant from day 1, and those of the wild-type increased over time, while the mutant remained nearly constant. This result suggests that the *rpoN* mutant is deficient in swimming motility.

### RpoN Is Essential for Biofilm Formation

**FIGURE 2:**
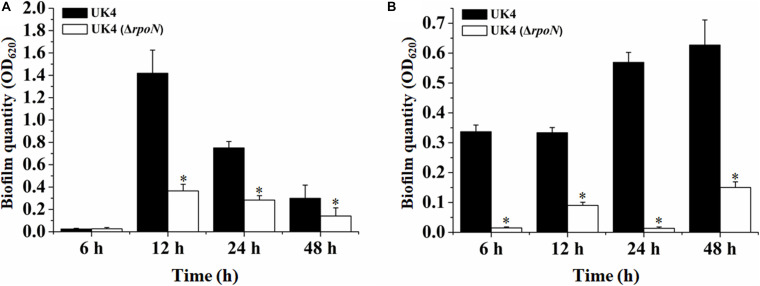
Biofilm biomass of *P*. *fluorescens* wild-type strain UK4 and the mutant strain Δ*rpoN* formed in microplates with LB medium **(A)** and tryptone broth **(B)**. The biofilm biomass was quantified by crystal violet staining. Data are expressed as means ± SD of eight replicates. Significant differences between UK4 and the mutant are analyzed by one-way ANOVA (analysis of variance). ^∗^*p* < 0.05.

Bacteria can adhere to solid surfaces to form biofilms that are extremely difficult to be detected and eradicated in the food industry (Winkelströter et al., 2014). To determine whether the *rpoN* gene is involved in the formation of solid surface-associated biofilms, the biofilm formations of UK4 and its *rpoN* mutant in plastic microplates were quantified through crystal violet staining. Two different media, LB and tryptone broth, were used in the experiments. As shown in Figure 2, the total biomass of the biofilm formed by the *rpoN* mutant was far below that of the wild-type in the two media, especially in tryptone broth.

**FIGURE 3:**
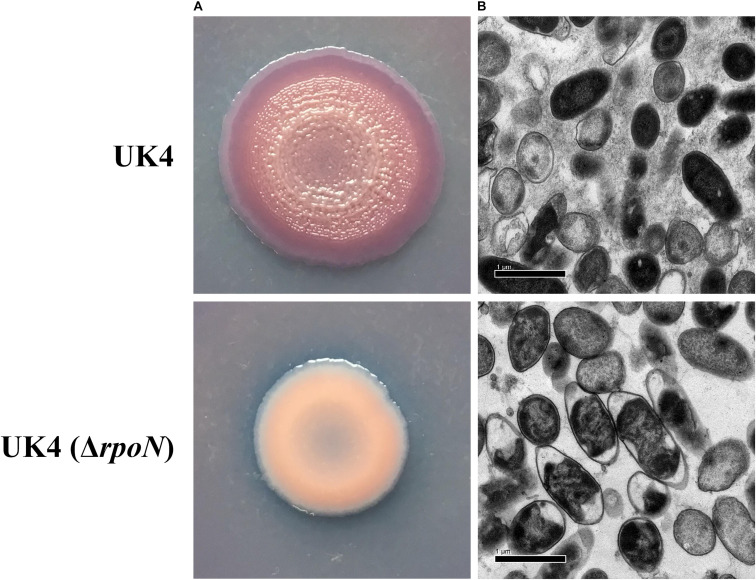
Macrocolony biofilm properties of the *P*. *fluorescens* wild-type strain UK4 and the mutant strain Δ*rpoN*. **(A)** Macrocolony morphology grown on Congo red and Coomassie brilliant blue plates for 7 days. **(B)** Transmission electron micrographs of the macrocolony biofilms at ×10,000 magnification.

Macrocolony biofilms formed on agar plates reflect the conditions of biofilms that grow on organic substrates such as soil or human food (Serra and Hengge, 2014). Congo red assay was used to determine whether the *rpoN* gene regulated the production of an extracellular matrix of macrocolony biofilm. The red and wrinkled phenotype on Congo-red plates often depends on the bacteria ability to produce a biofilm matrix (Friedman and Kolter, 2004). UK4 and its *rpoN* mutant were grown on tryptone plates containing Congo red for extended times (3–7 days) to form macrocolonies. As shown in Figure 3, UK4 strains formed dark red and wrinkled macrocolonies, while the *rpoN* mutant strains formed pale pink and smooth macrocolonies after 7 days of culture. Additionally, the macrocolonies were visualized at the cellular level via TEM, which showed that the wild-type cells were embedded in an extracellular matrix, whereas the mutant cells almost did not generate any extracellular matrix. These results suggest that the *rpoN* gene was involved in the biofilm matrix production.

### RpoN Regulates Stress and Antibiotic Resistance

**FIGURE 4:**
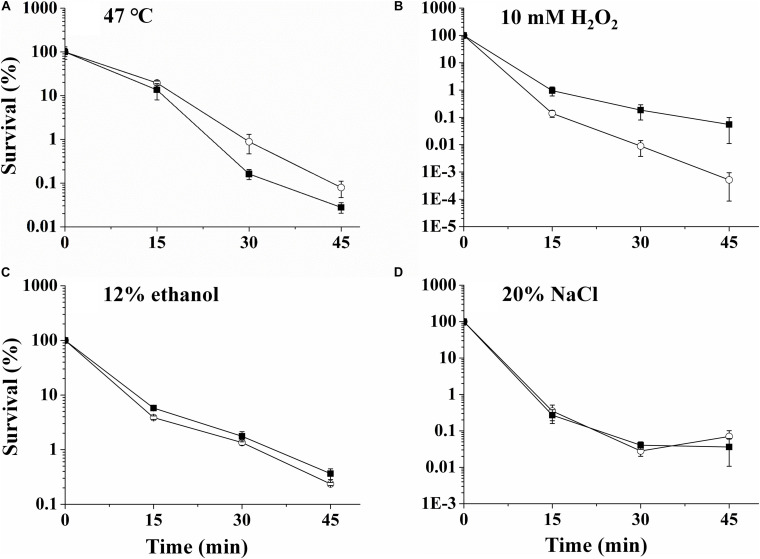
The survival of *P. fluorescens* wild-type strain UK4 (open circle) and the mutant strain Δ*rpoN* (closed diamond) after exposure to 47°C **(A)**, 10 mM H_2_O_2_ **(B)**, 12% ethanol **(C)**, and 20% NaCl **(D)**. Survival percentage was obtained by dividing the surviving population by the initial population, which corresponds to 100%. Data are expressed as mean ± SD of three independent experiments.

The stress resistances of UK4 and the *rpoN* mutant to diverse stress conditions, including exposures to 47°C, 10 mM H_2_O_2_, 12% ethanol, and 20% NaCl, were evaluated (Figure 4). During exposure to these conditions, the viabilities of the strains were determined by plate counts at 0, 15, 30, and 45 min. After exposure to 47°C, the survival rates of Δ*rpoN* cells were about 5.5 times and 2.8 times the values of UK4 at 30 and 45 min, respectively (Figure 4A). In contrast, after exposure to 10 mM H_2_O_2_, Δ*rpoN* cells showed survival rates of 6.8 times, 20.7 times, and 106.7 times those of UK4 at the time points 15, 30, and 45 min, respectively (Figure 4B). For the stress conditions of 12% ethanol and 20% NaCl, there was no significant difference between the survival rates of UK4 and Δ*rpoN* (*p* > 0.05) (Figures 4C,D).

The resistances of UK4 and the *rpoN* mutant to 19 antibiotics were determined by disk diffusion testing (Table 1). Compared with the wild-type, Δ*rpoN* was significantly more sensitive to nine antibiotics (*p* < 0.05): streptomycin, cefepime, rifampicin, nalidixic acid, fosfomycin, chloramphenicol, tetracycline, neomycin, and kanamycin. The wild-type and the mutant showed comparable susceptibilities to ciprofloxacin, norfloxacin, and gentamicin. In addition, both strains were not sensitive to seven other antibiotics: cefotaxime, sultamicillin, vancomycin, azithromycin, erythromycin, cephalexin, and penicillin.

**TABLE 1:** Results of antibiotic susceptibility of the wild-type and the *rpoN* mutant strains.

Antibiotics	The diameter of inhibitory zones (mm)	
	UK4	Δ*rpoN*
Streptomycin	20 ± 1	30 ± 1*
Cefepime	20 ± 2	25 ± 2*
Rifampicin	14 ± 1	21 ± 1*
Nalidixic acid	25 ± 2	36 ± 2*
Ciprofloxacin	33 ± 2	36 ± 2
Cefotaxime	–	–
Sultamicillin	–	–
Fosfomycin	22 ± 2	52 ± 1*
Chloramphenicol	20 ± 2	30 ± 1*
Vancomycin	–	–
Norfloxacin	40 ± 1	42 ± 2
Azithromycin	–	–
Erythromycin	–	–
Tetracycline	30 ± 2	42 ± 1*
Neomycin	15 ± 1	18 ± 1*
Kanamycin	30 ± 2	40 ± 1*
Gentamicin	20 ± 1	22 ± 1
Cephalexin	–	–
Penicillin	–	–

*p < 0.05.

*–, not sensitive.*

*Significance was analyzed with one-way ANOVA.*

### RpoN Positively Regulates Spoilage Potential of *P*. *fluorescens* in Sterile Fish Juice

**FIGURE 5:**
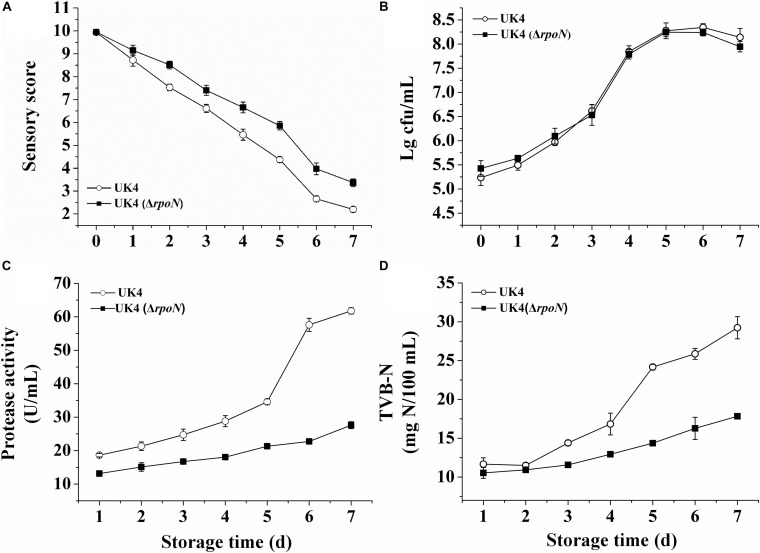
Sensory assessment **(A)**, TVC **(B)**, extracellular protease activity **(C)**, and TVB-N **(D)** of *P*. *fluorescens* wild-type strain UK4 and the mutant strain Δ*rpoN* in sterilized fish juice stored at 4°C. Data are expressed as mean ± SD of three independent experiments.

The spoilage potentials of UK4 and the *rpoN* mutant were compared in sterilized fish juice stored at 4°C by determining the sensory value, TVC, extracellular protease activity and TVB-N (Figure 5). The fish juice inoculated with the mutant showed significantly higher sensory scores and better characteristics for appearance and odor than that with the wild-type strain from day 2 (*p* < 0.05) (Figure 5A). According to the TVC, there was no significant difference between the growths of the wild-type and the mutant in sterilized fish juice stored at 4°C (*p* > 0.05). The two strains reached a stationary phase after 5 days of storage, with a cell population of more than 10^8^ cfu/mL (Figure 5B). Extracellular protease helps to decompose proteins in fish muscle, therefore, it is an important spoilage factor. As shown in Figure 5C, the extracellular protease activities of the mutant were always significantly lower than those of the wild-type during the storage time (*p* < 0.05), and the difference between them increased over time. On day 6, the activity of the wild-type increased remarkably, and reached about 2.5 times that of the mutant. In addition, the samples inoculated with UK4 or Δ*rpoN* showed low production of TVB-N and presented no significant difference in the initial 2 days (*p* > 0.05). By day 3, the TVB-N values of UK4 were significantly higher than those of Δ*rpoN*. The TVB-N values in the samples with UK4 were 68.2, 59.0, and 63.9% higher than those in the samples with *rpoN* mutant on day 5, day 6, and day 7, respectively. The results indicate that RpoN positively regulated the spoilage potential of *P*. *fluorescens* in sterilized fish juice stored at 4°C.

### Identification of RpoN-Regulated Genes by RNA-seq

**FIGURE 6:**
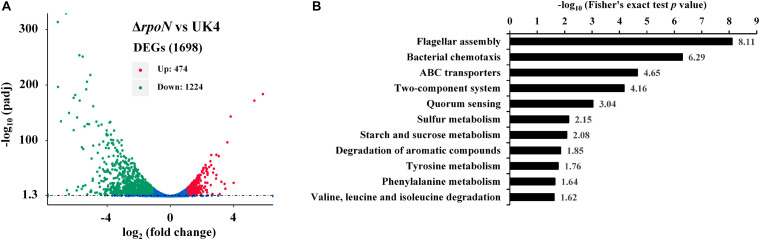
Results from RNA-seq analysis. **(A)** DEGs between the *rpoN* mutant and the wild-type strain. The horizontal axis represents fold changes of gene expression, and the vertical axis represents the statistically significant level. The red dots represent significantly upregulated genes, and the green dots represent significantly downregulated genes. **(B)** Results of KEGG pathway enrichment analysis of downregulated genes in the *rpoN* mutant. Significantly enriched pathways (*p* < 0.05) are shown in the figure.

**FIGURE 7:**
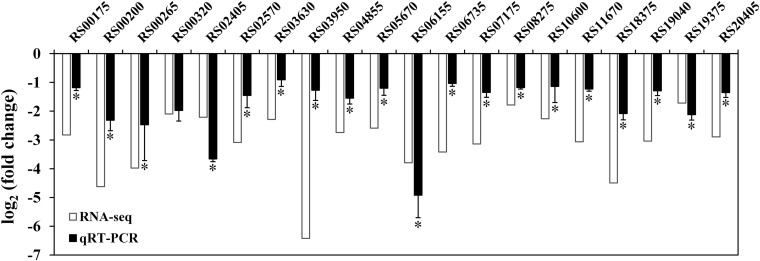
Confirmation of RNA-seq results using qRT-PCR. White bars represent RNA-seq data, while the black bars represent the mean values of log_2_ (fold change) obtained from the *rpoN* mutant samples compared with the wild-type samples. Data are presented as the mean ± SD (*n* = 6). Significant differences between the values of RNA-seq and qRT-PCR are analyzed by one-sample *t*-test. ^∗^*p* < 0.05.

To further investigate the regulatory role of RpoN, an RNA-seq dependent transcriptomics analysis was utilized to obtain RpoN-regulated genes. The *rpoN* mutant was compared with the wild-type strain, and three RNA-Seq libraries were prepared for each strain cultured in fish juice at 4°C for 6 days. Among different culturing times, we chose 6 days to perform the RNA-seq analysis because of the similar growth and great difference in spoilage potential between the mutant and the wild-type at this time point (Figure 5B). The raw sequencing data of RNA-seq were deposited in the Sequence Read Archive^2^, with accession numbers PRJNA663039. After the raw reads were filtered, an average of 15,187,604 clean reads for UK4 and 14,833,201 for the mutant were generated, resulting in an average 2.28 G and 2.22 G of total clean bases, respectively (Supplementary Table 3). The clean reads were mapped to the genome sequence of UK4, with the unique mapped rates of at least 98.38% for all samples (Supplementary Table 4). According to the screening criteria for DEGs (|log_2_ fold change| ≥ 1, padj ≤ 0.05), a total of 1698 DEGs were identified, including 1224 significantly downregulated genes and 474 significantly upregulated genes in the *rpoN* mutant compared with the wild-type (Figure 6A); this suggests that RpoN significantly functioned as a positive regulator in transcription. The detailed information on the downregulated and upregulated genes is summarized in Supplementary Tables 5, 6. The KEGG pathway enrichment analysis of the downregulated genes was performed. Eleven pathways were significantly enriched, including flagellar assembly, bacterial chemotaxis, starch and sucrose metabolism, valine, leucine, and isoleucine degradation (Figure 6B). To further confirm the downregulated genes from RNA-seq results, 20 downregulated genes were randomly selected to verify their expression via qRT-PCR (Figure 7). Although the fold change values were different, the qRT-PCR results agreed with the RNA-seq data, indicating that the RNA-seq results are reliable.

## Discussion

The results of this study illustrated the involvement of RpoN in regulating the swimming motility, biofilm formation, resistance to stress conditions and antibiotics, and spoilage activity of *P*. *fluorescens*. In addition, the RpoN-regulated genes were identified by the RNA-seq analysis of the *rpoN* mutant and the wild-type in fish juice stored at 4°C. The representative DEGs, including the genes related to flagellar mobility, adhesion, polysaccharide metabolism, resistance, amino acid transport and metabolism, and some other important genes, are listed in Table 2 for further discussion.

**TABLE 2:** Representative genes differentially expressed in the *rpoN* mutant compared with the wild-type strain UK4.

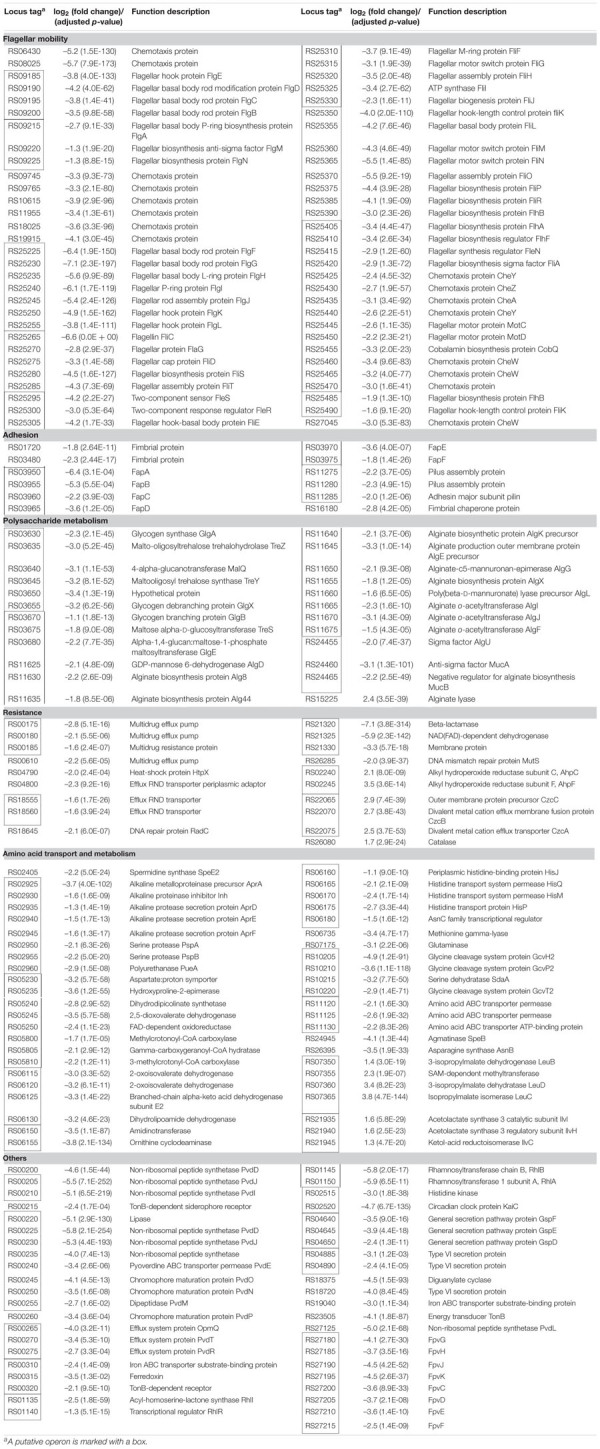

RpoN plays a great role in the regulation of flagellar biosynthesis and cell mobility in many bacteria (Francke et al., 2011). In our study, the Δ*rpoN* mutant was deficient in swimming motility. The RNA-seq results showed that dozens of downregulated genes in the Δ*rpoN* mutant were significantly enriched in flagellar assembly and bacterial chemotaxis pathways, suggesting that RpoN regulates swimming motility by controlling the expression of genes related to flagellar biosynthesis and chemotaxis (Figure 6B). In UK4, these genes were mainly located in two gene clusters (RS09185–RS09225, RS25225–RS25490) (Table 2), including flagellar structural genes (*flgBCDE*, *flgA, flgN*, *flgFGHIJKL*, *fliCflaGfliDfliSfliT*, *fliEFGHIJ*, *fliKMNOPQRflhB*, *flhA*, *motCD*), several regulatory protein genes (*flgM*, *fleSR*, *flhFfleNfliA*), and some genes encoding chemotaxis proteins. The expression of these genes are regulated by RpoN, which is consistent with the case in *P*. *aeruginosa* (Dasgupta et al., 2003).

Our results indicate that *P*. *fluorescens* can form robust biofilms on solid surfaces and semisolid agar plates, and RpoN was required in the biofilm formation (Figures 2, 3). As mentioned above, RpoN positively regulated the flagellar motility of *P*. *fluorescens*. This finding is consistent with the reports that the flagella mutants of *P*. *aeruginosa* PAO1 were deficient in biofilm formation in the wells of microplates when grown in minimal medium with glucose and casamino acids (Klausen et al., 2003). Therefore, RpoN may regulate the biofilm formation by controlling the flagellar motility in *P*. *fluorescens* UK4. However, Shao et al. (2018) found that the *rpoN* mutant of *P*. *aeruginosa* PAO1 increased the biofilm formation and reduced motility production. Biofilm formation is a complicated process, and it may be affected by other factors besides flagellar.

Bacterial fimbriae or pili are required for stable cell-to-surface adhesion and biofilm matrix formation (Muhammad et al., 2020). According to our RNA-seq results, several genes related to adhesion were notably downregulated in the *rpoN* mutant (Table 2), such as the *fapABCDEF* operon, related to amyloid-like fimbriae formation; the RS11285-75 operon coding for pilus formation; two genes (RS01720 and RS03480) encoding fimbrial protein; and one gene (RS16180) encoding fimbrial chaperone protein. An early paper reported similar results that RpoN was required for pilin formation in *P*. *aeruginosa* PAK (Ishimoto and Lory, 1989). However, Flp/Tad-T4b pili and Csu-T1 pili were proved to be negatively regulated by RpoN in *Pseudomonas protegens* H78 (Liu Y. et al., 2018). In the current work, RpoN positively regulated the *fap* operon. The Fap fibers were first identified in the *P*. *fluorescens* UK4 biofilm matrix, and the overexpression of the *fap* operon in *E*. *coli* resulted in a highly aggregative phenotype, showing that the expression of *fap* operon promotes biofilm formation (Dueholm et al., 2010). In addition, it has been well verified that in many other bacteria, fimbriae or pili play roles in surface adhesion, cell-cell aggregation, and biofilm formation (Muhammad et al., 2020). Therefore, in this work, the biofilm deficiency of Δ*rpoN* mutant may be due to the downregulation of genes related to fimbria or pilus formation.

Several downregulated genes were significantly enriched in the starch and sucrose metabolism pathway (Figure 6B), mainly located in two adjacent and reversed operons (RS03630-55 and RS03680-70) (Table 2). The genes *glgA*, *glgX*, *glgB*, and *glgE* are related to glycogen biosynthesis, while the gene *malQ* encoding the catabolic enzyme is related to glycogen catabolism. In addition, the three genes *treZ*, *treY*, and *treS* encode enzymes involved in producing trehalose by glycogen degradation. These genes were downregulated in the *rpoN* mutant, suggesting that RpoN positively regulated glycogen and trehalose metabolism. The homologs of these genes were also positively regulated by RpoN in the *kinB* mutant of *P*. *aeruginosa* PAO1 (Damron et al., 2012). In *E*. *coli*, the biofilm formation was improved by the expression of either the glycogen biosynthetic genes (*glgA* and *glgC*) or the glycogen catabolism gene (*glgP*) (Jackson et al., 2002). Quilès and Humbert (2014) found that the glycogen acted as an extracellular polymeric substance and participated in the spatial arrangement of the biofilm in *P*. *fluorescens* CIP 69.13 by attenuated total reflection Fourier transform infrared (ATR-FTIR) spectroscopy. Therefore, in the present work, the downregulation of the glycogen metabolism genes may also result in biofilm deficiency in the *rpoN* mutant. In addition, two operons *algD-F* (RS11625-75) and *algUmucAB* (RS24455-65), associated with alginate biosynthesis and regulation, respectively, were significantly downregulated in the *rpoN* mutant, and a gene encoding alginate lyase was significantly upregulated in the mutant (Table 2). Alginate is a significant polysaccharide of the biofilms produced by *P*. *aeruginosa* (Hentzer et al., 2001), and *rpoN* was required for high P*algU* and P*algD* promoter activities of this strain (Damron and Goldberg, 2012). Taken together, in our work RpoN may positively regulate the biofilm formation by controlling polysaccharide glycogen and alginate metabolism in *P*. *fluorescens*.

The resistances of *P*. *fluorescens* UK4 and its *rpoN* mutant to different stress conditions and multiple antibiotics were determined. The *rpoN* deletion decreased the resistance to 47°C, increased the resistance to H_2_O_2_, and did not influence the resistance to ethanol and NaCl. In addition, RpoN positively regulated the resistance of *P*. *fluorescens* to nine antibiotics. Thus, RpoN plays an important role in the resistance formation of *P*. *fluorescens*. As expected, several DEGs from RNA-seq may be responsible for the resistance variation (Table 2). The downregulation of genes coding for the heat-shock protein HtpX, DNA repair proteins, multidrug efflux pumps and beta-lactamase may cause reduced resistance of the *rpoN* mutant to heat stress and antibiotics. The membrane-bound heat shock protease HtpX plays a role in the removal of misfolded proteins under heat stress and contributes to heat resistance (Yoshitani et al., 2019). Heat or antibiotics can also cause DNA damage (Kohanski et al., 2007; Kantidze et al., 2016), and the damage may be reduced by DNA repair proteins, such as RadC or MutS. Resistance-nodulation-division (RND) transporters function as major drug efflux pumps in many Gram-negative bacteria and mainly contribute to the resistance to antimicrobial agents (Colclough et al., 2020). However, we found that the *rpoN* mutant was more resistant to H_2_O_2_. According to the RNA-seq results, several genes related to hyperoxide elimination were upregulated in the *rpoN* mutant, including AhpCF and catalase (Wan et al., 2019), and these enzymes may contribute to the H_2_O_2_ resistance of the *rpoN* mutant. RpoN has been shown to be important for stress and antibiotic resistance in other bacteria. For example, blocking RpoN has been found to increase susceptibility to several beta-lactam-based antibiotics in a laboratory strain of *P*. *aeruginosa* (Lloyd et al., 2019). The *rpoN* mutant of *Campylobacter jejuni* was more susceptible to acid stress; more resistant to H_2_O_2_; and had little effect on the resistance to alkaline pH, heat, cold, and antimicrobials than the wild-type strain (Hwang et al., 2011). Moreover, the effects of RpoN on stress and antibiotic resistance are not always the same among different species, which may be because the composition of the RpoN regulon differs substantially among different species.

The production of extracellular proteases by spoilage bacteria accelerates the degradation of protein in the food matrix, and amino acid degradation produces ammonia and biogenic amines with unpleasant and unacceptable off-flavors, which is a major cause of food spoilage (Ghaly et al., 2010; Polêto et al., 2019). Moreover, we determined the spoilage activities of UK4 and Δ*rpoN* in sterilized fish juice stored at 4°C. The *rpoN* deletion only had little effects on the TVC, but significantly reduced the production of extracellular protease and TVB-N. As expected, the RNA-seq results showed that many genes related to amino acid transport and metabolism were regulated by RpoN (Table 2). The following genes were significantly downregulated in the *rpoN* mutant: the operon *aprA*-*pueA* coding for biosynthesis and secretion of extracellular protease; the genes RS02405, RS05230-50, RS06150-55, RS06160-80, and RS24945, which are involved in arginine and proline metabolism and can lead to generation of putrescine, spermidine, and spermine besides ammonia; the operons RS05800-10 and RS06115-30 related to valine, leucine and isoleucine degradation; and the operon RS10205-20 related to glycine, serine and threonine degradation. In addition to the genes positively regulate by RpoN, the two operons RS07365-50 and RS21935-45 associated with valine, leucine, and isoleucine biosynthesis were negatively regulated by RpoN. These results indicate that RpoN mainly controls the spoilage activity of *P*. *fluorescens* by participating in the degradation and utilization of amino acids. Similarly, RpoN promotes extracellular protease secretion and arginine catabolism in *P*. *aeruginosa* and *E*. *coli* (Reitzer, 2003; Lloyd et al., 2017). Our previous studies showed that the *rpoS* mutant of *P*. *fluorescens* also reduced the production of extracellular protease and TVB-N in sterilized fish juice to some extent. In this work, we showed that RpoN is another important regulator controlling spoilage phenotypes of *P*. *fluorescens*.

Through RNA-seq analysis, we found that many other genes were also significantly downregulated in the *rpoN* mutant (Table 2), including genes related to the quorum-sensing system (RhlRI) and rhamnolipid biosurfactant synthesis (RhlAB), signal transduction, secretion system, and the biosynthesis and uptake of pyoverdine (Pvd and Fpv proteins). In addition, many genes with unknown function were noticeably regulated by RpoN, which will be further studied.

In conclusion, an in-frame deletion mutation of *rpoN* in *P*. *fluorescens* was constructed in this work to explore its function through phenotypic analysis and RNA-seq. Our results indicate that RpoN plays a great regulatory role in the swimming motility, biofilm formation, resistance to stress conditions and antibiotics, and spoilage potential in fish juice by controlling the expression of a large set of genes; these genes mainly include those related to flagellar mobility, adhesion, polysaccharide metabolism, resistance, and amino acid transport and metabolism. These findings reveal that RpoN is a global regulator of the spoilage activities of *P*. *fluorescens*. Moreover, RpoN and the RpoN-regulated pathways may serve as potential molecular targets for screening new food preservatives, or as microbial molecular markers to monitor food quality and safety.

## Data Availability Statement

The datasets presented in this study can be found in online repositories. The names of the repository/repositories and accession number(s) can be found in the article/Supplementary Material.

## Author Contributions

XL conceived and designed the experiments, performed the experiments, analyzed the data, and wrote the manuscript. YY and YZ performed the experiments. LW and LY took part in analyzing the RNA-seq data. JZ and AS took part in designing the experiments, interpreting the data, and revising the manuscript. XL, JZ, and AS functioned as co-correspondence. All the authors have read and approved the final version of the manuscript.

## Conflict of Interest

The authors declare that the research was conducted in the absence of any commercial or financial relationships that could be construed as a potential conflict of interest.

## Footnotes

1 http://www.kegg.jp/kegg/pathway.html

2 https://www.ncbi.nlm.nih.gov/sra/

## Abbreviations

ANOVA: analysis of variance
cfu: colony forming units
DAP: diaminopimelic acid
DEGs: differentially expressed genes
LB: Luria-Bertani
KEGG: Kyoto encyclopedia of genes and genomes
ORF: open reading frame
qRT-PCR: quantitative realtime PCR
RND: resistance-nodulation-division
RNA-seq: RNA sequencing
TEM: transmission electron microscopy
TVB-N: total volatile basic nitrogen
TVC: total viable count.
