# Supplementary material for: Involvement of RpoN in Regulating Motility, Biofilm, Resistance, and Spoilage Potential of Pseudomonas fluorescens
Source: Front Microbiol. 2021 May 31;12:641844. doi: 10.3389/fmicb.2021.641844 (PMC8202526; doi:10.3389/fmicb.2021.641844)
Supplement: Supplementary file 1 [file Table_1.DOCX]

**Supplementary Table S1** Strains, plasmids and primers used in this study.

| **Strains or plasmids** | **Characteristics** | **Reference or source** |
| --- | --- | --- |
| *Escherichia coli* |  |  |
| DH5α | F^-^, φ80d*lacZ*ΔM15, Δ(*lacZYA*-*argF*)U169, *deoR*, *recA*1, *endA*1, *hsdR*17(rk^-^, mk^+^), *phoA*, *supE*44, λ^-^, *thi*-1, *gyrA*96, *relA*1 | Invitrogen |
| β2163 | F^-^, RP4-2-Tc::Mu Δ*dapA*::(erm-pir) | Demarre et al. (2005) |
| *Pseudomonas fluorescens* |  |  |
| UK4 | Isolated from drinking water reservoir | the German Collection of  Microorganisms and Cell Cultures (DSMZ) |
| Δ*rpoN* | UK4, in-frame deletion of *rpoN* | This study |
| Plasmids |  |  |
| pLP12Tc | *oriT*_RP4_ *oriV*_R6K_ *vmi*480 P_BAD_, Tc^r^ | Laboratory collection |
| pLP12Tc-*rpoN* | pLP12Tc derivative containing *rpoN* bp 1-3 fused in-frame to bp 1474-1494, Tc^r^ | This study |
| Primers | Sequence (5’ - 3’) |  |
| For *rpoN* deletion |  |  |
| *rpoN*-MF1 | GGAATCTAGACCTTGAGTCGTCTCGGTAGGCGACATCAAGC | This study |
| *rpoN*-MR1 | GTCACATCAACCGCTTACGCTCCATGGCAGGGGCTTAACACC | This study |
| *rpoN*-MF2 | GGTGTTAAGCCCCTGCCATGGAGCGTAAGCGGTTGATGTGAC | This study |
| *rpoN*-MR2 | ACAGCTAGCGACGATATGTCTGAGGCGGCAATGAGGAATC | This study |
| *rpoN*-TF | GGAGTTCCACATCAACCATATTCG | This study |
| *rpoN*-TR | GCAGCACAAACAGCAGGTCAAC | This study |
