# Supplementary material for: Involvement of RpoN in Regulating Motility, Biofilm, Resistance, and Spoilage Potential of Pseudomonas fluorescens
Source: Front Microbiol. 2021 May 31;12:641844. doi: 10.3389/fmicb.2021.641844 (PMC8202526; doi:10.3389/fmicb.2021.641844)
Supplement: Supplementary file 2 [file Table_2.DOCX]

**Supplementary Table S2** List of primers used for qRT-PCR.

| Gene | Gene product description | Primer Sequences (5'to3') |
| --- | --- | --- |
| HZ99_RS00175 | multidrug efflux pump | F: GCGCACCTTGTTCCTGATG |
|  |  | R: GTTGACGTCGCCTGCAATCA |
| HZ99_RS00200 | non-ribosomal peptide synthetase PvdD | F: GCCTTCGGTACTGGTGTTGA |
|  |  | R: GCATACACCGACAACACGATACT |
| HZ99_RS00265 | efflux system protein OpmQ | F: GGTGATATGGCTGTCGAAGGA |
|  |  | R: CTGGCCTGGAATCACTGGTT |
| HZ99_RS00320 | TonB-dependent receptor | F: CACTGGCACTCCAACATATTCAC |
|  |  | R: GCATTGTCGCCCTCAACCA |
| HZ99_RS02405 | spermidine synthase SpeE2 | F: GGCACCAAAGCCGAGGAA |
|  |  | R: GCGATCAAGCCGATCCTGAT |
| HZ99_RS02570 | acyl-homoserine lactone acylase subunit beta | F: GCCAGTTCCTTGCCATTCATG |
|  |  | R: GTTCAAGGTACTTGAGGCAGAACA |
| HZ99_RS03630 | glycogen synthase GlgA | F: CCTGGGTGAGGTCAAATACG |
|  |  | R: CGGTCATCCATTGCTGGACTT |
| HZ99_RS03950 | FapA | F: GAGCGTCCCCTACTACCA |
|  |  | R: GCCAGATCTGCAGCTCTTC |
| HZ99_RS04855 | type IV secretion protein Rhs | F: GCCTCGCTGGAACTGCTA |
|  |  | R: GCGATGTGTTCGCACCAGA |
| HZ99_RS05670 | sugar ABC transporter permease | F: CCCAGCCGTGACCAGTT |
|  |  | R: CCAGCAGCCGTCCTTCAA |
| HZ99_RS06155 | ornithine cyclodeaminase | F: GTCTCGGATGCCTCGTTGT |
|  |  | R: GAATGCCATGACGGTGAGCAT |
| HZ99_RS06735 | methionine gamma-lyase | F: CGGTGCCTTGAATGTGTACTG |
|  |  | R: CCCAGGCTCAGCACATG |
| HZ99_RS07175 | glutaminase | F: GACACGCACCAGGGTGTA |
|  |  | R: CAGACCACGGTAAGCAAGCA |
| HZ99_RS08275 | nitrate transporter | F: GGCCATGTAGGGAAACTCGAA |
|  |  | R: CGCCGATGGACTCATCCTT |
| HZ99_RS10600 | TonB-dependent receptor | F: CAGCGAAACGGTTGTAGGCAAT |
|  |  | R: GGGCATCATGAACTCATTCC |
| HZ99_RS11670 | alginate o-acetyltransferase AlgJ | F: CAGCCTGGGGTTGATTTTCTC |
|  |  | R: CGCTGAAATAGGCCTTGAGGTA |
| HZ99_RS18375 | diguanylate cyclase | F: CCGAGATTGCGTTGGTCACT |
|  |  | R: GGTTACCACGTGGTCTGGAA |
| HZ99_RS19040 | iron ABC transporter substrate-binding protein | F: GCAACCTCGACCGTACAC |
|  |  | R: GGCCTGGCTGATGTCGTT |
| HZ99_RS19375 | nitrogen regulation protein NR(I) | F: CCACTCCCCGTCCTTATG |
|  |  | R: CTGACCCCTTGCTACAACGAT |
| HZ99_RS20405 | urease accessory protein UreG | F: CGTACCAGCGAGCGAATG |
|  |  | R: CCCGTTACCCACGAAGCA |
| 16S | internal control | F: GTCTCCTTAGAGTGCCCACCATTAC |
|  |  | R: GGTGCCTTCGGGAACATTGAGAC |
