# Supplementary material for: Involvement of RpoN in Regulating Motility, Biofilm, Resistance, and Spoilage Potential of Pseudomonas fluorescens
Source: Front Microbiol. 2021 May 31;12:641844. doi: 10.3389/fmicb.2021.641844 (PMC8202526; doi:10.3389/fmicb.2021.641844)
Supplement: Supplementary file 7 [file Data_Sheet_1.PDF]

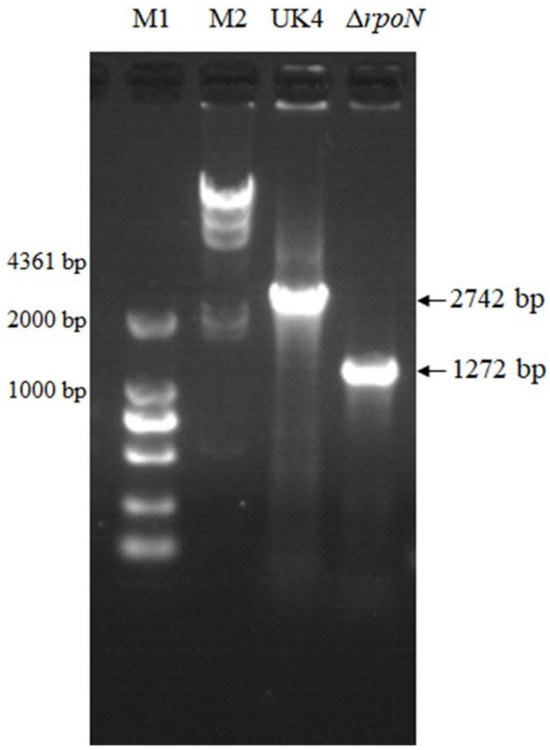

Supplementary Figure S1 PCR determination of *rpoN* in-frame deletion mutant of *P. fluorescens* UK4. Lane M1, DL2000 DNA Ladder; Lane M2, λ DNA/*Hind* III DNA Marker; Lane 1, wild-type strain UK4; Lane 2, *rpoN* in-frame deletion mutant.
